# (3-Acetyl-5-carboxyl­ato-4-methyl-1*H*-pyrazol-1-ido-κ^2^ *N* ^1^,*O* ^5^)aqua­[(pyridin-2-yl)methanamine-κ^2^ *N*,*N*′]copper(II)

**DOI:** 10.1107/S1600536812044959

**Published:** 2012-11-03

**Authors:** Sergey Malinkin, Vadim A. Pavlenko, Elzbieta Gumienna-Kontecka, Elena V. Prisyazhnaya, Turganbay S. Iskenderov

**Affiliations:** a Department of Chemistry, Kyiv National Taras Shevchenko University, Volodymyrska Str. 64, 01601 Kiev, Ukraine; b Faculty of Chemistry, University of Wrocław, F. Joliot-Curie Str. 14, 50-383, Wrocław, Poland; c Department of Chemistry, Kyiv National University of Construction and Architecture, Povitroflotsky Avenue 31, 03680 Kiev, Ukraine

## Abstract

In the title compound, [Cu(C_7_H_6_N_2_O_3_)(C_6_H_8_N_2_)(H_2_O)], the Cu^II^ ion is in a distorted square-pyramidal N_3_O_2_ environment formed by two bidentate chelating ligands in the equatorial coordination sites and one water mol­ecule in the apical direction. In the crystal, O—H⋯O, N—H⋯O and O—H⋯N hydrogen bonds link the complex mol­ecules into a three-dimensional supra­molecular network.

## Related literature

For applications of related pyrazoles, see: Sachse *et al.* (2008 ▶); Penkova *et al.* (2009 ▶). For synthetic and structural studies of 3,5-disubstituted 1*H*-pyrazoles and their metal complexes, see: Malinkin *et al.* (2011 ▶, 2012 ▶). For related structures, see: Fritsky *et al.* (2004 ▶); Kanderal *et al.* (2005 ▶); Krämer & Fritsky (2000 ▶); Moroz *et al.* (2010 ▶); Sliva *et al.* (1997 ▶); Wörl *et al.* (2005*a* ▶,*b* ▶).
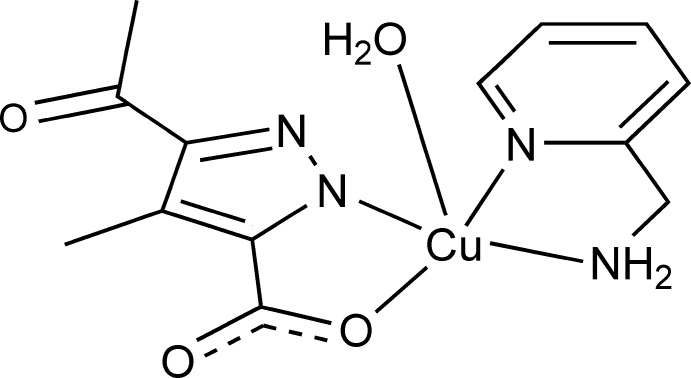


## Experimental

### 

#### Crystal data

[Cu(C_7_H_6_N_2_O_3_)(C_6_H_8_N_2_)(H_2_O)]
*M* *_r_* = 355.84
Triclinic, 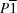

*a* = 7.3063 (2) Å
*b* = 8.3258 (5) Å
*c* = 13.1260 (7) Å
α = 90.695 (6)°
β = 105.935 (4)°
γ = 110.232 (4)°
*V* = 715.32 (6) Å^3^
*Z* = 2
Mo *K*α radiation
μ = 1.55 mm^−1^
*T* = 120 K
0.36 × 0.23 × 0.13 mm

#### Data collection

Nonius KappaCCD diffractometer
Absorption correction: multi-scan (*DENZO*/*SCALEPACK*; Otwinowski & Minor, 1997 ▶) *T*_min_ = 0.955, *T*_max_ = 0.987
13527 measured reflections
5715 independent reflections
4833 reflections with *I* > 2σ(*I*)
*R*_int_ = 0.016

#### Refinement

*R*[*F* ^2^ > 2σ(*F* ^2^)] = 0.027
*wR*(*F* ^2^) = 0.074
*S* = 1.07
5715 reflections
217 parameters
H atoms treated by a mixture of independent and constrained refinement
Δρ_max_ = 0.72 e Å^−3^
Δρ_min_ = −0.32 e Å^−3^

### 

Data collection: *COLLECT* (Nonius, 2000 ▶); cell refinement: *DENZO*/*SCALEPACK* (Otwinowski & Minor, 1997 ▶); data reduction: *DENZO*/*SCALEPACK*; program(s) used to solve structure: *SIR2004* (Burla *et al.*, 2005 ▶); program(s) used to refine structure: *SHELXL97* (Sheldrick, 2008 ▶); molecular graphics: *DIAMOND* (Brandenburg, 2009 ▶); software used to prepare material for publication: *SHELXL97*.

## Supplementary Material

Crystal structure: contains datablock(s) I, global. DOI: 10.1107/S1600536812044959/xu5636sup1.cif

Structure factors: contains datablock(s) I. DOI: 10.1107/S1600536812044959/xu5636Isup2.hkl

Additional supplementary materials:  crystallographic information; 3D view; checkCIF report

## Figures and Tables

**Table 1:** Selected bond lengths (Å)

Cu1—N1	1.9451 (9)
Cu1—N3	1.9973 (9)
Cu1—N4	2.0048 (10)
Cu1—O1	1.9874 (8)
Cu1—O4	2.3492 (8)

**Table 2:** Hydrogen-bond geometry (Å, °)

*D*—H⋯*A*	*D*—H	H⋯*A*	*D*⋯*A*	*D*—H⋯*A*
O4—H1*O*4⋯O2^i^	0.78 (2)	1.90 (2)	2.6782 (11)	176 (2)
O4—H2*O*4⋯N2^ii^	0.717 (18)	2.049 (18)	2.7581 (12)	169.7 (19)
N4—H1*N*4⋯O4^ii^	0.849 (17)	2.055 (17)	2.8542 (12)	156.6 (16)
N4—H2*N*4⋯O1^iii^	0.84 (2)	2.50 (2)	3.1017 (13)	128.6 (16)

Symmetry codes: (i) 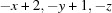; (ii) 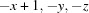; (iii) 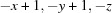.

**Fig. 1.**
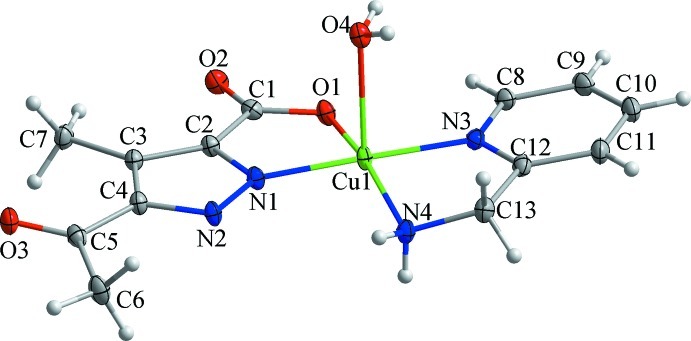
A view of the title compound, with displacement ellipsoids shown at the 50% probability level. H atoms are drawn as spheres of arbitrary radii.

**Fig. 2.**
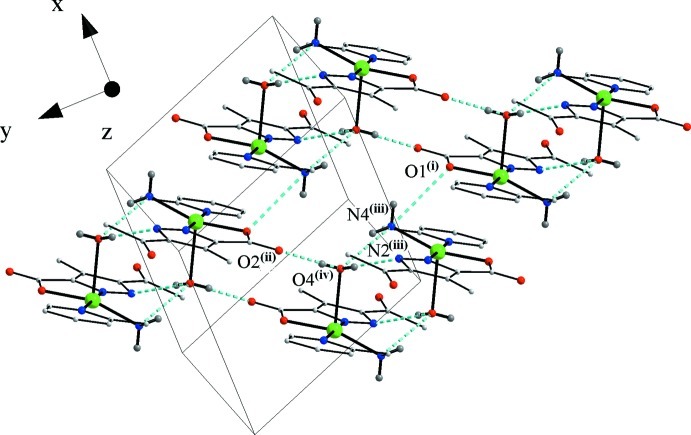
Crystal packing of the title compound. Hydrogen bonds are indicated by dashed lines. H atoms not involved in H-bonds are omitted for clarity. Symmetry codes: (i) -1 + x, -1 + y, z; (ii) 1 - x, 1 - y, -z; (iii) -x, -y, -z; (iv) -1 + x, y, z.

## supplementary crystallographic information

### Comment

Pyrazole–derived ligands are widely used in molecular magnetism, bioinorganic modelling and supramolecular chemistry due to their bridging nature and possibility for easy functionalization (Sachse *et al.*, 2008; Penkova *et al.*, 2009). Although usually this family of ligands is used for preparation of polynuclear complexes and coordination polymers, the mononuclear complexes based on pyrazole ligands can also represent an evident interest, especially as building block for preparation of polynuclear species. Herein we report the molecular and crystal structures of the title compound (Fig. 1) obtained in the framework of our synthetic and structural study of unsymmetrical 3,5-disubstituted pyrazolate ligands (Malinkin *et al.*, 2011, 2012).

The title compound, [Cu(C_6_H_6_N_2_O_3_)(C_6_H_8_N_2_)(H_2_O)] is a mononuclear mixed ligand complex, in which Cu^II^ ion is in distorted square-pyramidal environment formed by two bidentate (N, O) and (N, N) chelating ligands occupying four equatorial coordination sites and by the apically coordinated water molecule. While 2-aminomethylpyridine acts as a neutral ligand, the (3-acetyl-5-carboxylate)pyrazole ligand is a doubly charged acidoligand exhibiting its traditional (N, O)-chelating binding mode. The equatorial Cu—N and Cu—O bond lengths are in the range 1.9451 (9)–2.0048 (10) Å, whereas the apical Cu—O contact with water molecule is longer (2.3492 (8) Å). The coordination bond lengths Cu—N and Cu—O are typical for square-pyramidal Cu(II) complexes with the amine, deprotonated pyrazolate and carboxylate donors (Sliva *et al.*, 1997; Kanderal *et al.*, 2005). The bite angles around the central atom deviate from an ideal square-planar configuration [*e.g.* N1—Cu1—O2 = 82.74 (3)°], which is a consequence of the formation of five-membered chelate rings.

The C—N and C—C bond lengths in the pyridine rings are normal for 2-substituted pyridine derivatives (Krämer *et al.*, 2000; Moroz *et al.*, 2010). The C—C, C—N and N—N bond lengths in the pyrazole ring have their typical values (Sachse *et al.*, 2008; Penkova *et al.*, 2009). The C—O bond lengths in the deprotonated carboxylic groups differs significantly (1.2376 (13) and 1.2917 (13)) which is typical for monodentately coordinated carboxylates (Fritsky *et al.*, 2004; Wörl *et al.*, 2005*a*,*b*).

Numerous intermolecular O—H···O, N—H···O and O—H···N H-bonds in which the water molecules and the amine groups act as donors while the carboxylic groups, the water oxygen and the pyrazole nitrogen atoms act as acceptors unite the complex molecules in three-dimensional H-bonded network (Fig. 2).

### Experimental

To the solution of [Cu_4_(KA)_4_(H_2_O)_4_] *x* 4H_2_O (Malinkin *et al.*, 2011) (0.100 g, 0.078 mmol) in methanol (8 ml), 2-aminomethylpyridine (0.042 g, 0.391 mmol) was added. The reaction mixture was stirred upon ambient temperature for 10 minutes. Blue crystals suitable for X-ray diffraction were formed upon slow diffusion of diethyl ester into methanolic solution in 24 h (yield 0.028 g, 20%). Elemental analysis calc. (%) for C_13_H_18_CuN_4_O_4_: C 43.63; H 5.07; N 15.66; found: C 44.11; H 5.40; N 15.43.

### Refinement

The OH and NH hydrogen atoms were located from the difference Fourier map, and their positional and isotropic thermal parameters were included into the further stages of refinement. The C—H hydrogen atoms were positioned geometrically and were constrained to ride on their parent atoms, with C—H = 0.95–0.97 Å, and *U*_iso_ = 1.2–1.5 *U*_eq_(parent atom).

### Crystal data

**Table:**

[Cu(C_7_H_6_N_2_O_3_)(C_6_H_8_N_2_)(H_2_O)]	*Z* = 2
*M**_r_* = 355.84	*F*(000) = 366
Triclinic, *P*1	*D*_x_ = 1.652 Mg m^−^^3^
Hall symbol: -P 1	Mo *K*α radiation, λ = 0.71073 Å
*a* = 7.3063 (2) Å	Cell parameters from 2567 reflections
*b* = 8.3258 (5) Å	θ = 3.0–28.5°
*c* = 13.1260 (7) Å	µ = 1.55 mm^−^^1^
α = 90.695 (6)°	*T* = 120 K
β = 105.935 (4)°	Block, blue
γ = 110.232 (4)°	0.36 × 0.23 × 0.13 mm
*V* = 715.32 (6) Å^3^	

### Data collection

**Table:**

Nonius KappaCCD diffractometer	5715 independent reflections
Radiation source: fine-focus sealed tube	4833 reflections with *I* > 2σ(*I*)
Horizontally mounted graphite crystal monochromator	*R*_int_ = 0.016
Detector resolution: 9 pixels mm^-1^	θ_max_ = 35.1°, θ_min_ = 2.9°
φ scans and ω scans with κ offset	*h* = −11→9
Absorption correction: multi-scan (*DENZO*/*SCALEPACK*; Otwinowski & Minor, 1997)	*k* = −13→13
*T*_min_ = 0.955, *T*_max_ = 0.987	*l* = −20→21
13527 measured reflections	

### Refinement

**Table:**

Refinement on *F*^2^	Primary atom site location: structure-invariant direct methods
Least-squares matrix: full	Secondary atom site location: difference Fourier map
*R*[*F*^2^ > 2σ(*F*^2^)] = 0.027	Hydrogen site location: inferred from neighbouring sites
*wR*(*F*^2^) = 0.074	H atoms treated by a mixture of independent and constrained refinement
*S* = 1.07	*w* = 1/[σ^2^(*F*_o_^2^) + (0.0477*P*)^2^] where *P* = (*F*_o_^2^ + 2*F*_c_^2^)/3
5715 reflections	(Δ/σ)_max_ = 0.004
217 parameters	Δρ_max_ = 0.72 e Å^−^^3^
0 restraints	Δρ_min_ = −0.32 e Å^−^^3^

### Special details

**Table:**

Geometry. All s.u.'s (except the s.u. in the dihedral angle between two l.s. planes) are estimated using the full covariance matrix. The cell s.u.'s are taken into account individually in the estimation of s.u.'s in distances, angles and torsion angles; correlations between s.u.'s in cell parameters are only used when they are defined by crystal symmetry. An approximate (isotropic) treatment of cell s.u.'s is used for estimating s.u.'s involving l.s. planes.
Refinement. Refinement of *F*^2^ against ALL reflections. The weighted *R*-factor *wR* and goodness of fit *S* are based on *F*^2^, conventional *R*-factors *R* are based on *F*, with *F* set to zero for negative *F*^2^. The threshold expression of *F*^2^ > 2σ(*F*^2^) is used only for calculating *R*-factors(gt) *etc*. and is not relevant to the choice of reflections for refinement. *R*-factors based on *F*^2^ are statistically about twice as large as those based on *F*, and *R*- factors based on ALL data will be even larger.

### Fractional atomic coordinates and isotropic or equivalent isotropic displacement parameters (Å^2^)

**Table:**

	*x*	*y*	*z*	*U*_iso_*/*U*_eq_	
Cu1	0.545220 (19)	0.299111 (15)	0.020497 (9)	0.01226 (4)	
O1	0.74298 (12)	0.49534 (9)	−0.02392 (6)	0.01456 (14)	
O2	0.85228 (12)	0.57781 (10)	−0.16483 (6)	0.01670 (15)	
O3	0.22559 (13)	−0.03669 (11)	−0.47062 (6)	0.01945 (16)	
O4	0.79221 (13)	0.17036 (10)	0.06402 (6)	0.01427 (14)	
N1	0.45340 (14)	0.21529 (11)	−0.13062 (7)	0.01267 (15)	
N2	0.30428 (14)	0.08145 (11)	−0.19556 (7)	0.01319 (16)	
N3	0.63606 (14)	0.39053 (11)	0.17463 (7)	0.01323 (15)	
N4	0.29685 (15)	0.14866 (12)	0.05729 (7)	0.01486 (16)	
C1	0.73803 (15)	0.47312 (13)	−0.12234 (8)	0.01244 (17)	
C2	0.57544 (15)	0.31343 (12)	−0.18524 (8)	0.01178 (17)	
C3	0.50547 (16)	0.24117 (13)	−0.29113 (8)	0.01245 (17)	
C4	0.33336 (16)	0.09471 (13)	−0.29373 (8)	0.01263 (17)	
C5	0.19154 (17)	−0.03454 (13)	−0.38437 (8)	0.01427 (18)	
C6	0.00133 (18)	−0.16234 (15)	−0.36737 (9)	0.0198 (2)	
H6A	−0.0784	−0.2390	−0.4318	0.030*	
H6B	−0.0776	−0.1019	−0.3486	0.030*	
H6C	0.0386	−0.2276	−0.3108	0.030*	
C7	0.58880 (17)	0.30559 (15)	−0.38093 (9)	0.0172 (2)	
H7A	0.7016	0.4131	−0.3560	0.026*	
H7B	0.4839	0.3224	−0.4374	0.026*	
H7C	0.6345	0.2227	−0.4070	0.026*	
C8	0.79957 (17)	0.53218 (14)	0.22340 (9)	0.01563 (18)	
H8	0.8738	0.6015	0.1826	0.019*	
C9	0.86018 (18)	0.57730 (15)	0.33271 (9)	0.0189 (2)	
H9	0.9714	0.6771	0.3649	0.023*	
C10	0.75138 (19)	0.47038 (16)	0.39326 (9)	0.0204 (2)	
H10	0.7917	0.4959	0.4670	0.024*	
C11	0.58221 (19)	0.32535 (15)	0.34283 (9)	0.0187 (2)	
H11	0.5067	0.2531	0.3821	0.022*	
C12	0.52770 (17)	0.28997 (14)	0.23287 (8)	0.01449 (18)	
C13	0.34690 (17)	0.13433 (14)	0.17266 (9)	0.01577 (19)	
H13A	0.3780	0.0310	0.1864	0.019*	
H13B	0.2302	0.1252	0.1970	0.019*	
H1N4	0.236 (3)	0.048 (2)	0.0232 (14)	0.025 (4)*	
H2O4	0.773 (3)	0.114 (2)	0.1040 (14)	0.024 (4)*	
H2N4	0.207 (3)	0.194 (3)	0.0389 (16)	0.040 (5)*	
H1O4	0.895 (3)	0.247 (3)	0.0914 (17)	0.041 (5)*	

### Atomic displacement parameters (Å^2^)

**Table:**

	*U*^11^	*U*^22^	*U*^33^	*U*^12^	*U*^13^	*U*^23^
Cu1	0.01549 (7)	0.00997 (6)	0.00858 (6)	0.00179 (4)	0.00302 (4)	−0.00059 (4)
O1	0.0177 (4)	0.0105 (3)	0.0116 (3)	0.0019 (3)	0.0025 (3)	0.0001 (2)
O2	0.0156 (4)	0.0145 (3)	0.0161 (4)	0.0008 (3)	0.0045 (3)	0.0034 (3)
O3	0.0233 (4)	0.0230 (4)	0.0110 (3)	0.0081 (3)	0.0041 (3)	−0.0014 (3)
O4	0.0171 (4)	0.0098 (3)	0.0128 (3)	0.0018 (3)	0.0037 (3)	0.0014 (2)
N1	0.0140 (4)	0.0102 (4)	0.0107 (4)	0.0014 (3)	0.0029 (3)	0.0003 (3)
N2	0.0157 (4)	0.0109 (4)	0.0094 (4)	0.0016 (3)	0.0023 (3)	−0.0001 (3)
N3	0.0147 (4)	0.0134 (4)	0.0118 (4)	0.0059 (3)	0.0033 (3)	−0.0005 (3)
N4	0.0167 (4)	0.0139 (4)	0.0116 (4)	0.0033 (3)	0.0037 (3)	−0.0019 (3)
C1	0.0123 (4)	0.0106 (4)	0.0132 (4)	0.0039 (3)	0.0022 (3)	0.0015 (3)
C2	0.0128 (4)	0.0099 (4)	0.0110 (4)	0.0026 (3)	0.0030 (3)	0.0010 (3)
C3	0.0137 (4)	0.0133 (4)	0.0102 (4)	0.0047 (3)	0.0037 (3)	0.0015 (3)
C4	0.0155 (4)	0.0120 (4)	0.0097 (4)	0.0041 (3)	0.0036 (3)	0.0014 (3)
C5	0.0168 (5)	0.0124 (4)	0.0122 (4)	0.0055 (3)	0.0019 (4)	−0.0002 (3)
C6	0.0207 (5)	0.0167 (5)	0.0146 (5)	0.0008 (4)	0.0015 (4)	−0.0017 (3)
C7	0.0186 (5)	0.0204 (5)	0.0125 (4)	0.0050 (4)	0.0070 (4)	0.0023 (3)
C8	0.0142 (4)	0.0163 (5)	0.0149 (5)	0.0055 (4)	0.0021 (4)	−0.0029 (3)
C9	0.0171 (5)	0.0204 (5)	0.0164 (5)	0.0074 (4)	0.0001 (4)	−0.0055 (4)
C10	0.0227 (5)	0.0260 (6)	0.0117 (5)	0.0112 (4)	0.0012 (4)	−0.0034 (4)
C11	0.0230 (5)	0.0229 (5)	0.0117 (5)	0.0097 (4)	0.0057 (4)	0.0009 (4)
C12	0.0182 (5)	0.0156 (4)	0.0119 (4)	0.0087 (4)	0.0047 (4)	0.0005 (3)
C13	0.0191 (5)	0.0152 (4)	0.0133 (4)	0.0055 (4)	0.0062 (4)	0.0017 (3)

### Geometric parameters (Å, º)

**Table:**

Cu1—N1	1.9451 (9)	C3—C7	1.4937 (15)
Cu1—N3	1.9973 (9)	C4—C5	1.4724 (15)
Cu1—N4	2.0048 (10)	C5—C6	1.5055 (16)
Cu1—O1	1.9874 (8)	C6—H6A	0.9600
Cu1—O4	2.3492 (8)	C6—H6B	0.9600
O1—C1	1.2917 (13)	C6—H6C	0.9600
O2—C1	1.2376 (13)	C7—H7A	0.9600
O3—C5	1.2252 (13)	C7—H7B	0.9600
O4—H2O4	0.717 (18)	C7—H7C	0.9600
O4—H1O4	0.78 (2)	C8—C9	1.3852 (15)
N1—N2	1.3351 (12)	C8—H8	0.9300
N1—C2	1.3558 (13)	C9—C10	1.3895 (18)
N2—C4	1.3622 (13)	C9—H9	0.9300
N3—C12	1.3422 (14)	C10—C11	1.3868 (17)
N3—C8	1.3473 (14)	C10—H10	0.9300
N4—C13	1.4736 (14)	C11—C12	1.3862 (15)
N4—H1N4	0.849 (17)	C11—H11	0.9300
N4—H2N4	0.84 (2)	C12—C13	1.5056 (15)
C1—C2	1.4842 (14)	C13—H13A	0.9700
C2—C3	1.3899 (14)	C13—H13B	0.9700
C3—C4	1.4087 (14)		
			
N1—Cu1—O1	82.74 (3)	C3—C4—C5	129.26 (9)
N1—Cu1—N3	178.33 (4)	O3—C5—C4	121.35 (10)
O1—Cu1—N3	96.71 (3)	O3—C5—C6	121.30 (10)
N1—Cu1—N4	97.67 (4)	C4—C5—C6	117.34 (9)
O1—Cu1—N4	162.48 (4)	C5—C6—H6A	109.5
N3—Cu1—N4	82.38 (4)	C5—C6—H6B	109.5
N1—Cu1—O4	94.29 (3)	H6A—C6—H6B	109.5
O1—Cu1—O4	88.99 (3)	C5—C6—H6C	109.5
N3—Cu1—O4	87.27 (3)	H6A—C6—H6C	109.5
N4—Cu1—O4	108.40 (4)	H6B—C6—H6C	109.5
C1—O1—Cu1	113.96 (6)	C3—C7—H7A	109.5
Cu1—O4—H2O4	109.7 (14)	C3—C7—H7B	109.5
Cu1—O4—H1O4	104.8 (15)	H7A—C7—H7B	109.5
H2O4—O4—H1O4	106 (2)	C3—C7—H7C	109.5
N2—N1—C2	110.24 (8)	H7A—C7—H7C	109.5
N2—N1—Cu1	136.81 (7)	H7B—C7—H7C	109.5
C2—N1—Cu1	112.89 (7)	N3—C8—C9	121.77 (11)
N1—N2—C4	106.46 (8)	N3—C8—H8	119.1
C12—N3—C8	119.53 (9)	C9—C8—H8	119.1
C12—N3—Cu1	114.19 (7)	C8—C9—C10	118.62 (11)
C8—N3—Cu1	126.10 (8)	C8—C9—H9	120.7
C13—N4—Cu1	110.41 (7)	C10—C9—H9	120.7
C13—N4—H1N4	109.1 (12)	C9—C10—C11	119.49 (10)
Cu1—N4—H1N4	116.1 (11)	C9—C10—H10	120.3
C13—N4—H2N4	110.5 (13)	C11—C10—H10	120.3
Cu1—N4—H2N4	107.6 (13)	C12—C11—C10	118.78 (11)
H1N4—N4—H2N4	102.8 (17)	C12—C11—H11	120.6
O2—C1—O1	124.10 (9)	C10—C11—H11	120.6
O2—C1—C2	120.82 (9)	N3—C12—C11	121.76 (10)
O1—C1—C2	115.01 (9)	N3—C12—C13	116.41 (9)
N1—C2—C3	109.44 (9)	C11—C12—C13	121.81 (10)
N1—C2—C1	114.77 (8)	N4—C13—C12	110.16 (9)
C3—C2—C1	135.63 (9)	N4—C13—H13A	109.6
C2—C3—C4	103.02 (9)	C12—C13—H13A	109.6
C2—C3—C7	128.53 (10)	N4—C13—H13B	109.6
C4—C3—C7	128.43 (9)	C12—C13—H13B	109.6
N2—C4—C3	110.82 (9)	H13A—C13—H13B	108.1
N2—C4—C5	119.92 (9)		
			
N1—Cu1—O1—C1	−6.64 (7)	O2—C1—C2—N1	−176.16 (10)
N3—Cu1—O1—C1	174.95 (7)	O1—C1—C2—N1	1.03 (13)
N4—Cu1—O1—C1	−99.11 (13)	O2—C1—C2—C3	−1.25 (19)
O4—Cu1—O1—C1	87.82 (7)	O1—C1—C2—C3	175.94 (11)
O1—Cu1—N1—N2	−176.05 (11)	N1—C2—C3—C4	0.34 (11)
N3—Cu1—N1—N2	−105.3 (12)	C1—C2—C3—C4	−174.76 (11)
N4—Cu1—N1—N2	−13.71 (11)	N1—C2—C3—C7	178.83 (10)
O4—Cu1—N1—N2	95.53 (11)	C1—C2—C3—C7	3.7 (2)
O1—Cu1—N1—C2	7.03 (7)	N1—N2—C4—C3	−0.11 (12)
N3—Cu1—N1—C2	77.8 (12)	N1—N2—C4—C5	−179.58 (9)
N4—Cu1—N1—C2	169.36 (7)	C2—C3—C4—N2	−0.14 (12)
O4—Cu1—N1—C2	−81.40 (7)	C7—C3—C4—N2	−178.64 (10)
C2—N1—N2—C4	0.33 (12)	C2—C3—C4—C5	179.26 (10)
Cu1—N1—N2—C4	−176.65 (8)	C7—C3—C4—C5	0.77 (19)
N1—Cu1—N3—C12	107.5 (12)	N2—C4—C5—O3	−172.17 (10)
O1—Cu1—N3—C12	178.03 (7)	C3—C4—C5—O3	8.47 (18)
N4—Cu1—N3—C12	15.67 (8)	N2—C4—C5—C6	8.75 (15)
O4—Cu1—N3—C12	−93.31 (8)	C3—C4—C5—C6	−170.61 (11)
N1—Cu1—N3—C8	−77.6 (12)	C12—N3—C8—C9	0.21 (16)
O1—Cu1—N3—C8	−7.01 (9)	Cu1—N3—C8—C9	−174.50 (8)
N4—Cu1—N3—C8	−169.37 (9)	N3—C8—C9—C10	1.62 (17)
O4—Cu1—N3—C8	81.65 (9)	C8—C9—C10—C11	−2.07 (18)
N1—Cu1—N4—C13	158.90 (7)	C9—C10—C11—C12	0.76 (18)
O1—Cu1—N4—C13	−110.94 (12)	C8—N3—C12—C11	−1.61 (16)
N3—Cu1—N4—C13	−22.79 (7)	Cu1—N3—C12—C11	173.71 (8)
O4—Cu1—N4—C13	61.76 (8)	C8—N3—C12—C13	179.85 (9)
Cu1—O1—C1—O2	−178.07 (8)	Cu1—N3—C12—C13	−4.84 (12)
Cu1—O1—C1—C2	4.84 (11)	C10—C11—C12—N3	1.12 (17)
N2—N1—C2—C3	−0.44 (12)	C10—C11—C12—C13	179.58 (10)
Cu1—N1—C2—C3	177.32 (7)	Cu1—N4—C13—C12	25.68 (10)
N2—N1—C2—C1	175.79 (8)	N3—C12—C13—N4	−14.04 (13)
Cu1—N1—C2—C1	−6.45 (11)	C11—C12—C13—N4	167.42 (10)

### Hydrogen-bond geometry (Å, º)

**Table:**

*D*—H···*A*	*D*—H	H···*A*	*D*···*A*	*D*—H···*A*
O4—H1*O*4···O2^i^	0.78 (2)	1.90 (2)	2.6782 (11)	176 (2)
O4—H2*O*4···N2^ii^	0.717 (18)	2.049 (18)	2.7581 (12)	169.7 (19)
N4—H1*N*4···O4^ii^	0.849 (17)	2.055 (17)	2.8542 (12)	156.6 (16)
N4—H2*N*4···O1^iii^	0.84 (2)	2.50 (2)	3.1017 (13)	128.6 (16)

Symmetry codes: (i) −*x*+2, −*y*+1, −*z*; (ii) −*x*+1, −*y*, −*z*; (iii) −*x*+1, −*y*+1, −*z*.
